# Predictive factors for latency period in viable pregnancies complicated by preterm premature rupture of the membranes

**DOI:** 10.4274/tjod.30643

**Published:** 2015-03-15

**Authors:** Cihan Çetin, Selim Büyükkurt, Ercan Cömert, Ferda Özlü, Nilgün Bahar, Cansun Demir

**Affiliations:** 1 Çukurova University Faculty of Medicine, Department of Obstetrics and Gynecology, Adana, Turkey; 2 Çukurova University Faculty of Medicine, Department of Neonatology, Adana, Turkey

**Keywords:** Cervical length measurement, fetal membranes premature rupture, infant, premature diseases, premature birth, pregnancy complications, infectious

## Abstract

**Objective:**

In this study, we aimed to evaluate some laboratory and clinical factors in the prediction of latency period for pregnant patients complicated with preterm premature rupture of the membranes.

**Materials and Methods:**

Sixty-five pregnant patients between 24 and 34 weeks of gestation, who were admitted to University of Çukurova School of Medicine Hospital with the diagnosis of preterm premature rupture of the membranes (PPROM) between January 01, 2013 and December 31, 2013, were included in this study. Serum CRP, procalcitonin, sedimentation rate, leukocyte count and cervical length (measured with transvaginal ultrasound) of patients were analyzed for the correlation with the latency period.

**Results:**

None of the parameters were found to be correlated with the latency period. However, patients with cervical length of <25 mm were found to have shorter duration of latency.

**Conclusion:**

Although preterm premature rupture of the membranes is thought to be either an infection-based disease or a disease increasing the risk of infectious complications, major infection markers are not found to be helpful criteria for the prediction of latency period. Patients with a cervical length of <25 mm can be expected to deliver earlier and, therefore, can be referred to a tertiary center earlier.

## INTRODUCTION

The term premature rupture of the membranes is defined as the leakage of the amniotic fluid before the onset of labor. If it occurs before 37 weeks of gestation, it is defined as preterm premature rupture of the membranes (PPROM). PPROM is estimated to occur in 2-3.5% of pregnancies^(1)^. It is an important obstetric problem, because it is related to serious maternal and fetal complications. These complications include chorioamnionitis, placental abruption, oligohydramnios, fetal pulmonary hypoplasia, fetal extremity contractures, umbilical cord prolapse, and stillbirth. Furthermore, PPROM also increases neonatal complications related to preterm birth and infections (neonatal sepsis). Therefore, the diagnosis and management of PPROM is of critical importance in order to avoid serious fetal, maternal and neonatal consequences^(2)^.

Several studies showed limited or no benefit of prolongation of pregnancies complicated by PPROM beyond 34 weeks of gestation^(3)^. Despite antibiotic and steroid therapies, no benefit for the fetus is expected, therefore, delivery should be offered to these pregnants in order to prevent potential maternal infectious complications^(4)^. However, pregnancies between 34 weeks and 24 weeks may benefit from conservative approach^(5)^. These patients should be given corticosteroids for lung maturation together with the use of empiric antibiotics for the prophylaxis against infectious complications.

During expectant management of these patients, currently, there is no clinical or laboratory parameter to project the spontaneous labor and delivery time, so called latency period. Determination of latency period is essential in patients with PPROM in order to initiate treatments, transport patient to an adequately equipped unit, and inform and organize neonatology team on time. In this study, we aimed to investigate the efficacy of various laboratory and clinical parameters in predicting the latency period for patients with PPROM.

## MATERIALS AND METHODS

In this retrospective study, we analyzed the data of 65 pregnant patients who were admitted to University of Çukurova School of Medicine Hospital with the diagnosis of PPROM between January 1, 2013 and December 31, 2013. We retrieved patients’ data from our institution’s archive records.

All patients were between 24 and 37 weeks of gestation. PPROM diagnosis was established by either the visualization of evident amniotic fluid passing from the cervical canal during speculum examination or by vaginal pH above 6 in the posterior fornix. In order to exclude false positive cases, patients, whose speculum examination revealed blood or semen contamination or bacterial vaginosis, were excluded from the study, unless evident amniotic fluid passing from cervical canal is seen. Multiple pregnancies and pregnancies with PPROM before 24 weeks of gestation were excluded from the study.

Patients’ serum parameters (CRP, leukocyte count, sedimentation rate, procalcitonin) and cervical length (measured by transvaginal ultrasound) were noted at the time of the initial diagnosis of PPROM.

All patients at or beyond 34 weeks of gestation were offered immediate delivery, but some (four patients) chose to proceed their pregnancy. Patients between 24 and 34 weeks of gestation were followed up after betamethasone administration. These patients were given amoxicillin and spiramycine for one week and then discharged without any treatment unless vaginal or urinary cultures are sensitive to empirically given antibiotics. They are closely followed up on outpatient basis until 34 weeks and delivered thereafter.

For this study, we got ethical approval from our institution’s local ethics committee. SPSS version 16 was used for statistical analyses (descriptive statistics, student’s t-test, ANOVA and correlation studies) and a p value of less than 0.05 was considered statistically significant.

## RESULTS

Demographic and clinical characteristics of patients and the number of patients with the most important risk factors for PPROM (prior PPROM, DM, polyhydramnios, smoking) are shown on Table 1. Median gravidity and parity of patients are shown on Tables 2 and 1, respectively.

Patients are divided into three groups according to their gestational age (24-28, 29-33 and 34-36). Mean latency periods and mean gestational ages at delivery in these groups are shown on Table 2.

Mean C-reactive protein (CRP), sedimentation rate, procalcitonin and leukocyte counts at the time of PPROM diagnosis is shown on Table 3. There was no statistically significant correlation between these laboratory parameters and the latency period in groups.

The mean cervical length, which was measured transvaginally, was 31.67±6.5 mm (20-41). There was no correlation between cervical length and the latency period (p>0.05). However, when cervical length cut off point is set at 25 mm and patients are divided into two groups (group 1: <25 mm and group 2: ≥25 mm), mean latency periods of groups were 0.14 and 3.36 weeks, respectively (p<0.05).

Twenty-seven (42%) patients delivered vaginally whereas 38 (59%) patients delivered by cesarean section (C/S). Cesarean indications were having previous C/S in 18 patients and for other obstetric indications in 20 patients. Sixty-two (95%) patients delivered live babies and 3 (5%) had stillbirth. Obstetric outcomes of patients are summarized on Table 4. Also, the mean Apgar scores of live born babies are shown on Table 5.

Thirty-three born babies (51%) were male and 32 were female (49%).

The total hospitalization days needed are given on Table 6. There was a statistically significant difference between the groups (p<0.001); most hospitalized patients were between 24-28 weeks, as expected.

One patient with PPROM at 26^th^ week developed chorioamnionitis at 3^th^ week during follow-up and immediately delivered thereafter with an Apgar score of 8/9. The newborn was hospitalized for 8 days and discharged without any morbidity.

## DISCUSSION

Main morbidity and mortality rates for pregnancies complicated by PPROM vary among different gestational ages. As gestational age advances, perinatal outcomes improve. While the worst perinatal outcome for pregnancies diagnosed with PPROM is expected to occur in pregnancies before viability (generally accepted as 24 weeks of gestation), the best outcome is expected after 34 weeks.

Although the causes of PPROM are multifactorial, infection has a leading role both as the cause and the consequence^(6)^. Ascending vaginal microorganisms secrete various enzymes that weaken the amniotic membrane and lead to PPROM. Initially, these microorganisms cause subclinical infections and later, these are followed by clinical infections such as chorioamnionitis and fetal infections. Thus, the latency period is thought to be prolonged by the antibiotic administration and this is found to be effective in various studies^(6,7,8)^.

PPROM is generally thought to be an infection-based disease. Serum markers of infectious diseases can therefore be expected to rise during the progression of the disease. The aim of this study was to test the efficacy of infection markers to help predict the level of the disease on route to delivery. Widely used infection markers such as leukocyte count, procalcitonin, sedimentation rate and CRP were used. However, we were unable to find a direct correlation between the levels of these markers and the latency period of PPROM.

Although procalcitonin was found to be increased in patients with PPROM, Torbe et al. did not find a direct correlation between serum procalcitonin levels and the latency period^(9,10)^. However, to the best of our knowledge, there is no study directly investigated the correlation of the levels of CRP, sedimentation and leukocytosis with the latency period. These parameters are shown to increase in patients with PPROM who are complicated by chorioamnionitis leading to immediate delivery^(10)^. One of our patients, who had PPROM at 26 weeks of gestation, developed chorioamnionitis after four weeks of a conservative follow-up. At the time of the diagnosis of chorioamnionitis, high CRP (24.6 mg/dl), leukocyte (22.000), sedimentation rate (100 mm/h) and procalcitonin (0.31) levels were found. Therefore, follow-up of these parameters may be helpful in the early diagnosis of chorioamnionitis.

Cervical length is another important prognostic parameter in the preterm delivery^(11)^. Cervical length did not correlate with the latency period in our study. However, when cervical length is subgrouped into two with the cut-off point 25 mm, we found significantly shorter latency periods in patients with less than 25 mm. Indeed, Fischer et al. also did not find correlation between cervical length (measured by translabial ultrasound) and latency period in their study^(12)^. However, in this study, a cervical length of less than 25 mm was not associated with a shorter latency period.

Limitation of our study is that, we offer immediate delivery to patients after 34 weeks of gestation. Therefore we cannot directly make correlation studies in this subgroup.

In conclusion, baseline maternal serum parameters (CRP, leukocyte count, sedimentation rate, procalcitonin) are not found to be helpful in predicting the latency period. Likewise, cervical length is not correlated with the latency period. However, not surprisingly, patients with PPROM and short cervix (less than 25 mm) can be expected to deliver earlier. Further studies with these and other parameters are needed to be conducted in order to successfully estimate the latency period in patients with PPROM.

## Figures and Tables

**Table 1:**
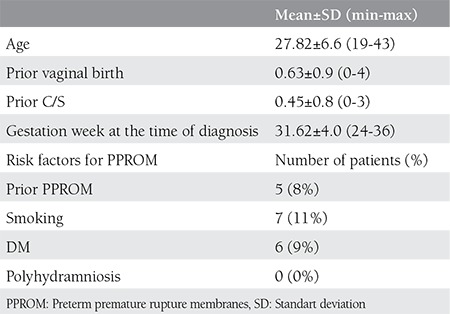
Demographic and clinical characteristics of patients and number of patients with risk factors for preterm premature rupture membranes (PPROM)

**Table 2:**
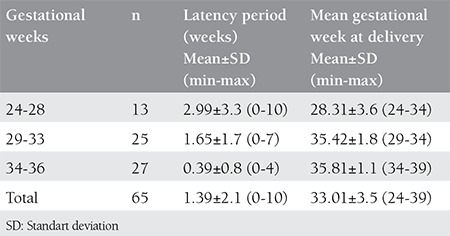
Mean latency periods and gestational weeks at delivery

**Table 3:**
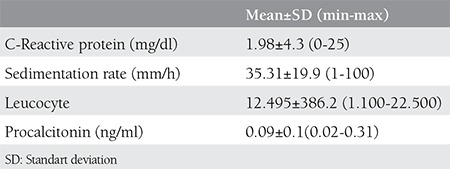
Mean levels of infection markers at the time of diagnosis

**Table 4:**
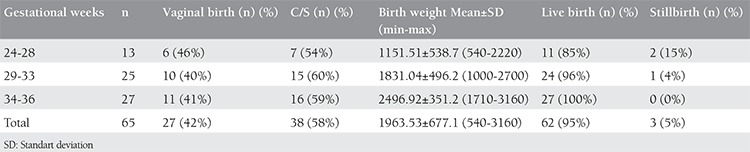
The obstetric outcomes of patients

**Table 5:**
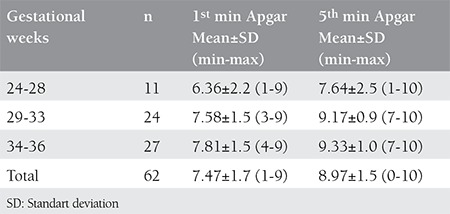
Mean Apgar scores of liveborn babies

**Table 6:**
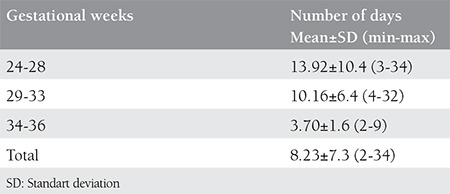
Total hospitalization days of patients
